# 4,4,5,5-Tetra­methyl-1,3,2λ^5^-dioxa­phospho­lan-2-one

**DOI:** 10.1107/S160053681102959X

**Published:** 2011-07-30

**Authors:** Anna Skarżyńska, Anna M. Trzeciak, Andrzej Gniewek

**Affiliations:** a Faculty of Chemistry, University of Wrocław, 14 F. Joliot-Curie, 50-383 Wrocław, Poland

## Abstract

The five-membered ring in the title compound, C_6_H_13_O_3_P, exists in an envelope conformation with one of the ring C atoms at the flap position. The coordination geometry around the P atom is a distorted tetra­hedron. The crystal structure is stabilized by several weak C—H⋯O and P—H⋯O hydrogen bonds, forming a three-dimensional network.

## Related literature

For a discussion of 1,3,2-dioxaphospho­lane chemistry, see: Maffei & Buono (2003 ▶); Zwierzak (1967 ▶) and for the Heck reaction, see: Beletskaya & Cheprakov (2000 ▶); Skarżyńska *et al.* (2011 ▶). For hydrogen-bond inter­actions, see: Desiraju & Steiner (1999 ▶). For bond lengths in organic compounds, see: Allen *et al.* (1987 ▶). For details of the temperature control applied during data collection, see: Cosier & Glazer (1986 ▶) and for specifications of the analytical numeric absorption correction, see: Clark & Reid (1995 ▶).
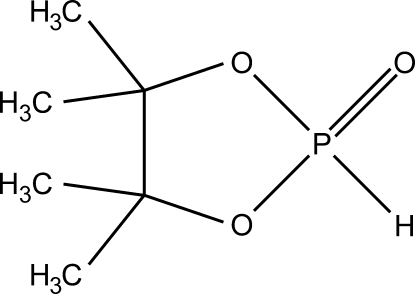


## Experimental

### 

#### Crystal data

C_6_H_13_O_3_P
*M* *_r_* = 164.13
Monoclinic, 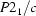

*a* = 7.144 (2) Å
*b* = 7.570 (2) Å
*c* = 15.064 (4) Å
β = 90.98 (2)°
*V* = 814.5 (4) Å^3^
*Z* = 4
Mo *K*α radiation
μ = 0.29 mm^−1^
*T* = 100 K
0.33 × 0.27 × 0.26 mm

#### Data collection

Kuma KM-4 diffractometer with CCD detector
Absorption correction: analytical (*CrysAlis RED*; Oxford Diffraction, 2010 ▶) *T*_min_ = 0.910, *T*_max_ = 0.952
7254 measured reflections
1869 independent reflections
1673 reflections with *I* > 2σ(*I*)
*R*_int_ = 0.021

#### Refinement

*R*[*F* ^2^ > 2σ(*F* ^2^)] = 0.035
*wR*(*F* ^2^) = 0.096
*S* = 1.10
1869 reflections
143 parameters
All H-atom parameters refined
Δρ_max_ = 0.49 e Å^−3^
Δρ_min_ = −0.30 e Å^−3^

### 

Data collection: *CrysAlis CCD* (Oxford Diffraction, 2010 ▶); cell refinement: *CrysAlis RED* (Oxford Diffraction, 2010 ▶); data reduction: *CrysAlis RED*; program(s) used to solve structure: *SHELXS97* (Sheldrick, 2008 ▶); program(s) used to refine structure: *SHELXL97* (Sheldrick, 2008 ▶); molecular graphics: *ORTEP-3* (Farrugia, 1997 ▶); software used to prepare material for publication: *SHELXL97*.

## Supplementary Material

Crystal structure: contains datablock(s) global, I. DOI: 10.1107/S160053681102959X/bt5583sup1.cif

Structure factors: contains datablock(s) I. DOI: 10.1107/S160053681102959X/bt5583Isup2.hkl

Supplementary material file. DOI: 10.1107/S160053681102959X/bt5583Isup3.cml

Additional supplementary materials:  crystallographic information; 3D view; checkCIF report

## Figures and Tables

**Table 1:** Hydrogen-bond geometry (Å, °)

*D*—H⋯*A*	*D*—H	H⋯*A*	*D*⋯*A*	*D*—H⋯*A*
C11—H113⋯O^i^	0.98 (2)	2.70 (2)	3.583 (2)	150 (2)
C12—H123⋯O^i^	1.00 (2)	2.60 (2)	3.499 (2)	150 (2)
C21—H213⋯O^i^	0.96 (2)	2.68 (2)	3.544 (2)	148 (2)
C22—H222⋯O1^ii^	0.98 (2)	2.64 (2)	3.515 (2)	148 (2)
P—H⋯O1^iii^	1.28 (2)	2.58 (2)	3.4713 (12)	124 (1)

Symmetry codes: (i) 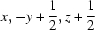; (ii) 
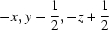
; (iii) 
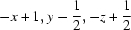
.

**Fig. 1.**
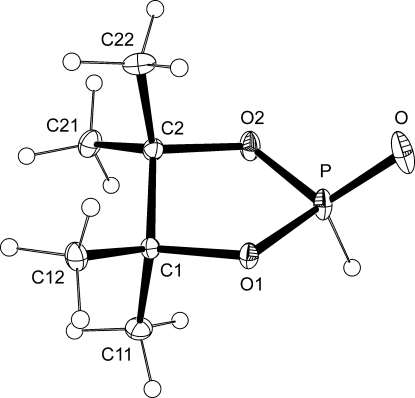
The molecular structure and atom numbering scheme of the title compound. Displacement ellipsoids are drawn at the 30% probability level and H atoms are shown as small spheres of arbitrary radii.

## supplementary crystallographic information

### Comment

Carbon-carbon bond-forming catalytic reactions are very important fundamental processes in synthetic chemistry. Among them, one of the commonly recognized is the Heck reaction employing as the catalyst precursors palladium compounds with phosphorus ligands (Beletskaya & Cheprakov, 2000). Complexes incorporating in their structure 1,3,2-dioxaphospholane heterocyclic rings have been recently found to be efficient catalysts of the Heck reaction carried out under mild conditions (Skarżyńska *et al.*, 2011). In this paper we report the synthesis and crystallization of tetramethyl dioxaphospholane, the title compound.

The geometric parameters around the four-coordinate phosphorus atom (Fig. 1) indicate a deformation of the ideal tetrahedron towards a trigonal pyramid. The O—P—O1 and O—P—O2 angles differ considerably from the ideal value of 109.5° and approach 120°, while O1—P—O2 is close to 90°. Such deformations might be explained by the effect of different substituents and bond types. Bond lengths P—O1, P—O2, P—O, and P—H are typical (Allen *et al.*, 1987). The heterocyclic five-membered ring P/O1/C1/C2/O2 adopts an envelope conformation with the C1 atom deviating from the four-atom plane by about 0.55 Å.

The crystal structure is stabilized by a few hydrogen bonds of the C—H···O and P—H···O types (Desiraju & Steiner, 1999). Consequently, a three-dimensional network of such interactions is formed in the crystal. The C11, C12 and C21 atoms act as hydrogen-bond donors, *via* H113, H123 and H213, respectively, to the O^i^ atom [symmetry code: (i) *x*, –*y* + 1/2, *z* + 1/2] as an acceptor (Table 1). As a result, chains running parallel to the [001] direction are formed. The adjacent chains of the molecules are further linked by C22—H222···O1^ii^ and P—H···O1^iii^ hydrogen interactions [symmetry codes: (ii) –*x*, *y* – 1/2, –*z* + 1/2; (iii) –*x* + 1, *y* – 1/2, –*z* + 1/2].

### Experimental

The title compound may be prepared according to the known procedures: utilizing pinacol and phosphorus trichloride, followed by hydrolysis (Zwierzak, 1967) or involving a transestrification process between pinacol and diethyl phosphite (Maffei & Buono, 2003). Here we report an alternative route. Crystallization of 2,2,3,3,7,7,8,8-octamethyl-1,4,6,9–5λ^5^-phosphaspiro[4.4]nonane in non-dried diethyl ether leads to hydrolysis of the tetraoxaspirophosphorane. As a result, 4,4,5,5-teramethyl-1,3,2-dioxaphospholane 2-oxide is formed as single crystals.

### Refinement

All H atoms were found in a difference Fourier map and refined isotropically. The measured C—H distances in methyl groups are in range 0.92 (2)–1.00 (2)Å and P—H bond length is 1.28 (2) Å. The highest residual peak and the deepest hole in the final difference map are located 0.77 and 0.76Å from the C1 and P atom, respectively.

### Crystal data

**Table:**

C_6_H_13_O_3_P	*F*(000) = 352
*M**_r_* = 164.13	*D*_x_ = 1.338 Mg m^−^^3^
Monoclinic, *P*2_1_/*c*	Mo *K*α radiation, λ = 0.71073 Å
Hall symbol: -P 2ybc	Cell parameters from 4820 reflections
*a* = 7.144 (2) Å	θ = 3.0–27.5°
*b* = 7.570 (2) Å	µ = 0.29 mm^−^^1^
*c* = 15.064 (4) Å	*T* = 100 K
β = 90.98 (2)°	Block, colorless
*V* = 814.5 (4) Å^3^	0.33 × 0.27 × 0.26 mm
*Z* = 4	

### Data collection

**Table:**

Kuma KM-4 diffractometer with CCD detector	1869 independent reflections
Radiation source: fine-focus sealed tube	1673 reflections with *I* > 2σ(*I*)
graphite	*R*_int_ = 0.021
ω scans	θ_max_ = 27.5°, θ_min_ = 3.0°
Absorption correction: analytical (*CrysAlis RED*; Oxford Diffraction, 2010)	*h* = −9→9
*T*_min_ = 0.910, *T*_max_ = 0.952	*k* = −9→9
7254 measured reflections	*l* = −19→13

### Refinement

**Table:**

Refinement on *F*^2^	Primary atom site location: structure-invariant direct methods
Least-squares matrix: full	Secondary atom site location: difference Fourier map
*R*[*F*^2^ > 2σ(*F*^2^)] = 0.035	Hydrogen site location: difference Fourier map
*wR*(*F*^2^) = 0.096	All H-atom parameters refined
*S* = 1.10	*w* = 1/[σ^2^(*F*_o_^2^) + (0.0645*P*)^2^ + 0.1298*P*] where *P* = (*F*_o_^2^ + 2*F*_c_^2^)/3
1869 reflections	(Δ/σ)_max_ < 0.001
143 parameters	Δρ_max_ = 0.49 e Å^−^^3^
0 restraints	Δρ_min_ = −0.30 e Å^−^^3^

### Special details

**Table:**

Experimental. The crystal was placed in the cold stream of an open-flow nitrogen cryostat (Cosier & Glazer, 1986) operating at 100 K. Analytical numeric absorption correction was carried out with *CrysAlis RED* (Oxford Diffraction, 2010) using a multifaceted crystal model (Clark & Reid, 1995).
Geometry. All e.s.d.'s (except the e.s.d. in the dihedral angle between two l.s. planes) are estimated using the full covariance matrix. The cell e.s.d.'s are taken into account individually in the estimation of e.s.d.'s in distances, angles and torsion angles; correlations between e.s.d.'s in cell parameters are only used when they are defined by crystal symmetry. An approximate (isotropic) treatment of cell e.s.d.'s is used for estimating e.s.d.'s involving l.s. planes.
Refinement. Refinement of *F*^2^ against ALL reflections. The weighted *R*-factor *wR* and goodness of fit *S* are based on *F*^2^, conventional *R*-factors *R* are based on *F*, with *F* set to zero for negative *F*^2^. The threshold expression of *F*^2^ > 2σ(*F*^2^) is used only for calculating *R*-factors(gt) *etc*. and is not relevant to the choice of reflections for refinement. *R*-factors based on *F*^2^ are statistically about twice as large as those based on *F* and *R*- factors based on ALL data will be even larger.

### Fractional atomic coordinates and isotropic or equivalent isotropic displacement parameters (Å^2^)

**Table:**

	*x*	*y*	*z*	*U*_iso_*/*U*_eq_	
P	0.35196 (5)	0.22996 (4)	0.21158 (2)	0.02085 (14)	
H	0.528 (2)	0.199 (2)	0.2076 (11)	0.028 (4)*	
O	0.26815 (19)	0.25962 (13)	0.12379 (7)	0.0376 (3)	
O1	0.32677 (12)	0.38610 (11)	0.28007 (5)	0.0172 (2)	
C1	0.29786 (16)	0.31569 (15)	0.37050 (8)	0.0152 (2)	
C11	0.48914 (18)	0.28153 (19)	0.41307 (10)	0.0236 (3)	
H111	0.553 (2)	0.190 (2)	0.3832 (12)	0.030 (4)*	
H112	0.563 (3)	0.389 (3)	0.4098 (12)	0.044 (5)*	
H113	0.476 (3)	0.251 (2)	0.4759 (14)	0.037 (5)*	
C12	0.19155 (18)	0.45553 (17)	0.42081 (8)	0.0213 (3)	
H121	0.076 (2)	0.494 (2)	0.3871 (11)	0.031 (4)*	
H122	0.265 (2)	0.555 (2)	0.4267 (10)	0.026 (4)*	
H123	0.163 (2)	0.408 (2)	0.4813 (11)	0.033 (4)*	
O2	0.27159 (13)	0.07451 (11)	0.26996 (6)	0.0224 (2)	
C2	0.18549 (16)	0.14270 (16)	0.35218 (8)	0.0168 (3)	
C21	0.21170 (19)	0.00213 (17)	0.42256 (9)	0.0235 (3)	
H211	0.339 (2)	−0.037 (2)	0.4228 (10)	0.028 (4)*	
H212	0.131 (3)	−0.095 (3)	0.4074 (12)	0.037 (5)*	
H213	0.179 (2)	0.044 (3)	0.4806 (12)	0.040 (5)*	
C22	−0.02050 (19)	0.1768 (2)	0.33204 (11)	0.0278 (3)	
H221	−0.039 (3)	0.273 (2)	0.2898 (13)	0.034 (5)*	
H222	−0.069 (2)	0.063 (3)	0.3105 (11)	0.040 (5)*	
H223	−0.082 (3)	0.215 (2)	0.3860 (13)	0.029 (4)*	

### Atomic displacement parameters (Å^2^)

**Table:**

	*U*^11^	*U*^22^	*U*^33^	*U*^12^	*U*^13^	*U*^23^
P	0.0308 (2)	0.0184 (2)	0.0136 (2)	0.00484 (12)	0.00665 (14)	0.00041 (11)
O	0.0656 (8)	0.0339 (6)	0.0132 (5)	0.0071 (5)	0.0000 (5)	−0.0014 (4)
O1	0.0224 (4)	0.0161 (4)	0.0131 (4)	0.0004 (3)	0.0037 (3)	0.0011 (3)
C1	0.0160 (5)	0.0173 (5)	0.0123 (5)	−0.0012 (4)	0.0002 (4)	0.0012 (4)
C11	0.0167 (6)	0.0305 (7)	0.0235 (7)	−0.0014 (5)	−0.0050 (5)	0.0027 (5)
C12	0.0271 (6)	0.0195 (6)	0.0175 (6)	0.0021 (5)	0.0032 (5)	−0.0034 (5)
O2	0.0343 (5)	0.0159 (4)	0.0171 (4)	0.0007 (4)	0.0071 (4)	−0.0023 (3)
C2	0.0175 (5)	0.0169 (5)	0.0162 (5)	−0.0005 (4)	0.0027 (4)	−0.0012 (4)
C21	0.0282 (6)	0.0194 (6)	0.0232 (7)	−0.0016 (5)	0.0061 (5)	0.0051 (5)
C22	0.0173 (6)	0.0290 (7)	0.0368 (8)	−0.0047 (5)	−0.0031 (5)	−0.0009 (6)

### Geometric parameters (Å, °)

**Table:**

P—O	1.4596 (12)	C12—H123	1.00 (2)
P—H	1.28 (2)	C1—C2	1.5582 (16)
P—O1	1.5812 (10)	O2—C2	1.4850 (14)
P—O2	1.5827 (10)	C2—C21	1.5115 (16)
O1—C1	1.4806 (14)	C2—C22	1.5196 (16)
C1—C12	1.5137 (16)	C21—H211	0.96 (2)
C1—C11	1.5216 (16)	C21—H212	0.96 (2)
C11—H111	0.95 (2)	C21—H213	0.96 (2)
C11—H112	0.98 (2)	C22—H221	0.98 (2)
C11—H113	0.98 (2)	C22—H222	0.98 (2)
C12—H121	1.00 (2)	C22—H223	0.97 (2)
C12—H122	0.92 (2)		
			
O—P—O1	115.26 (6)	H121—C12—H122	106 (2)
O—P—O2	118.08 (6)	H121—C12—H123	113 (2)
O—P—H	112.0 (8)	H122—C12—H123	109 (2)
O1—P—O2	98.44 (6)	C2—O2—P	111.37 (7)
O1—P—H	106.9 (8)	O2—C2—C21	106.99 (10)
O2—P—H	104.7 (8)	O2—C2—C22	107.82 (10)
C1—O1—P	110.52 (7)	C21—C2—C22	111.57 (10)
O1—C1—C11	108.09 (10)	O2—C2—C1	102.73 (9)
O1—C1—C12	106.74 (10)	C21—C2—C1	114.22 (10)
C12—C1—C11	111.25 (10)	C22—C2—C1	112.75 (10)
O1—C1—C2	102.66 (9)	C2—C21—H211	109.1 (12)
C11—C1—C2	112.83 (10)	C2—C21—H212	107.7 (12)
C12—C1—C2	114.52 (10)	C2—C21—H213	112.1 (12)
C1—C11—H111	111.0 (12)	H211—C21—H212	109 (2)
C1—C11—H112	108.7 (12)	H211—C21—H213	110 (2)
C1—C11—H113	110.5 (12)	H212—C21—H213	109 (2)
H111—C11—H112	109 (2)	C2—C22—H221	112.1 (12)
H111—C11—H113	110 (2)	C2—C22—H222	104.5 (12)
H112—C11—H113	108 (2)	C2—C22—H223	109.4 (12)
C1—C12—H121	111.4 (12)	H221—C22—H222	113 (2)
C1—C12—H122	109.0 (12)	H221—C22—H223	105 (2)
C1—C12—H123	108.5 (12)	H222—C22—H223	112 (2)
			
O—P—O1—C1	143.16 (9)	O1—C1—C2—O2	37.31 (10)
O2—P—O1—C1	16.56 (8)	C11—C1—C2—O2	−78.79 (12)
P—O1—C1—C11	85.34 (8)	C12—C1—C2—O2	152.58 (12)
P—O1—C1—C12	−154.89 (10)	O1—C1—C2—C21	152.79 (10)
P—O1—C1—C2	−34.11 (10)	C11—C1—C2—C21	36.69 (12)
O—P—O2—C2	−116.09 (9)	C12—C1—C2—C21	−91.94 (12)
O1—P—O2—C2	8.52 (8)	O1—C1—C2—C22	−78.47 (10)
P—O2—C2—C21	−149.19 (8)	C11—C1—C2—C22	165.44 (12)
P—O2—C2—C22	90.67 (10)	C12—C1—C2—C22	36.80 (12)
P—O2—C2—C1	−28.61 (10)		

### Hydrogen-bond geometry (Å, °)

**Table:**

*D*—H···*A*	*D*—H	H···*A*	*D*···*A*	*D*—H···*A*
C11—H113···O^i^	0.98 (2)	2.70 (2)	3.583 (2)	150 (2)
C12—H123···O^i^	1.00 (2)	2.60 (2)	3.499 (2)	150 (2)
C21—H213···O^i^	0.96 (2)	2.68 (2)	3.544 (2)	148 (2)
C22—H222···O1^ii^	0.98 (2)	2.64 (2)	3.515 (2)	148 (2)
P—H···O1^iii^	1.28 (2)	2.58 (2)	3.4713 (12)	124 (1)

Symmetry codes: (i) *x*, −*y*+1/2, *z*+1/2; (ii) −*x*, *y*−1/2, −*z*+1/2; (iii) −*x*+1, *y*−1/2, −*z*+1/2.
